# The Association of Low Vitamin K Status with Mortality in a Cohort of 138 Hospitalized Patients with COVID-19

**DOI:** 10.3390/nu13061985

**Published:** 2021-06-09

**Authors:** Allan Linneberg, Freja Bach Kampmann, Simone Bastrup Israelsen, Liv Rabøl Andersen, Henrik Løvendahl Jørgensen, Håkon Sandholt, Niklas Rye Jørgensen, Sanne Marie Thysen, Thomas Benfield

**Affiliations:** 1 Center for Clinical Research and Prevention, Copenhagen University Hospital—Bispebjerg and Frederiksberg, DK-2000 Copenhagen, Denmark; freja.bach.kampmann.02@regionh.dk (F.B.K.); sanne.marie.thysen.01@regionh.dk (S.M.T.); 2 Department of Clinical Medicine, Faculty of Health and Medical Sciences, University of Copenhagen, DK-2200 Copenhagen, Denmark; henrik.loevendahl.joergensen.01@regionh.dk (H.L.J.); niklas.rye.joergensen@regionh.dk (N.R.J.); thomas.lars.benfield@regionh.dk (T.B.); 3 Center of Research and Disruption of Infectious Diseases, Department of Infectious Diseases, Copenhagen University Hospital—Amager and Hvidovre, DK-2650 Hvidovre, Denmark; simone.elisabeth.bastrup.israelsen.02@regionh.dk (S.B.I.); liv.raboel.andersen@regionh.dk (L.R.A.); haakon.sandholdt@regionh.dk (H.S.); 4 Department of Clinical Biochemistry, Copenhagen University Hospital—Amager and Hvidovre, DK-2650 Hvidovre, Denmark; 5 Department of Clinical Biochemistry, Copenhagen University Hospital—Rigshospitalet, DK-2600 Glostrup, Denmark

**Keywords:** vitamin K, COVID-19, SARS-CoV-2, Matrix Gla Protein, elastic fiber, thrombosis

## Abstract

It has recently been hypothesized that vitamin K could play a role in COVID-19. We aimed to test the hypotheses that low vitamin K status is a common characteristic of patients hospitalized with COVID-19 compared to population controls and that low vitamin K status predicts mortality in COVID-19 patients. In a cohort of 138 COVID-19 patients and 138 population controls, we measured plasma dephosphorylated-uncarboxylated Matrix Gla Protein (dp-ucMGP), which reflects the functional vitamin K status in peripheral tissue. Forty-three patients died within 90 days from admission. In patients, levels of dp-ucMGP differed significantly between survivors (mean 877; 95% CI: 778; 995) and non-survivors (mean 1445; 95% CI: 1148; 1820). Furthermore, levels of dp-ucMGP (pmol/L) were considerably higher in patients (mean 1022; 95% CI: 912; 1151) compared to controls (mean 509; 95% CI: 485; 540). Cox regression survival analysis showed that increasing levels of dp-ucMGP (reflecting low vitamin K status) were associated with higher mortality risk (sex- and age-adjusted hazard ratio per doubling of dp-ucMGP was 1.49, 95% CI: 1.03; 2.24). The association attenuated and became statistically insignificant after adjustment for co-morbidities (sex, age, CVD, diabetes, BMI, and eGFR adjusted hazard ratio per doubling of dp-ucMGP was 1.22, 95% CI: 0.82; 1.80). In conclusion, we found that low vitamin K status was associated with mortality in patients with COVID-19 in sex- and age-adjusted analyses, but not in analyses additionally adjusted for co-morbidities. Randomized clinical trials would be needed to clarify a potential role, if any, of vitamin K in the course of COVID-19.

## 1. Introduction

Vitamin K is an essential vitamin for the activation of blood clotting factors and several other proteins, indicating that vitamin K has effects on coagulation, bone formation and the inhibition of calcification in arteries [1]. Vitamin K serves as a co-factor for the enzyme γ-glutamate carboxylase that converts glutamate residues into γ-carboxyglutamate (Gla). These Gla residues serve as calcium-binding groups, which are essential for the activity of all Gla-containing proteins. Vitamin K status can be objectively assessed in two different ways: (A) by measuring the vitamin K concentration in plasma or (B) by determining the amount of uncarboxylated vitamin K-dependent proteins. The first method reflects a snapshot of recent vitamin K intake, is sensitive to triglyceride concentrations, and gives limited information about vitamin K utilization in tissue. In contrast, the dephosphorylated-uncarboxylated isoform of MGP (dp-ucMGP) reflects the functional vitamin K status and is considered the gold standard for measuring vitamin K status in peripheral tissue [2,3].

Coronavirus disease 2019 (COVID-19) is a transmittable viral infection caused by Severe Acute Respiratory Syndrome Coronavirus-2 (SARS-CoV-2) [4]. COVID-19 presents very differently in patients; many patients experience mild symptoms, while other patients develop severe disease, including respiratory failure with high risk of death [5]. A recently published study found significantly higher levels of dp-ucMGP (reflecting lower vitamin K status) in hospitalized COVID-19 patients compared to controls, suggesting a role of vitamin K in COVID-19 [6].

Among COVID-19 patients, low vitamin K status was associated with a poor outcome of disease (need for invasive ventilation or death). Furthermore, low vitamin K status was associated with increased blood levels of desmosine, a biomarker of degradation of elastic fibers in the lung tissue [7], suggesting that low vitamin K status could increase the rate of degradation of elastic fibers during severe COVID-19. The authors hypothesized that increased degradation of elastic fibers in the lungs could be due to a lack of activated MGP, which is known to protect extracellular matrix proteins such as elastic fibers from calcification and subsequent degradation [8]. MGP is the strongest known inhibitor of tissue calcification in the arterial vessel wall and thus, prevents arterial calcification [7]. MGP is also highly expressed in the lungs [9,10]. Degradation of elastic fibers in the lungs stimulates calcification of elastic fibers [11]. A rising calcium content of the extracellular matrix stimulates the local synthesis of MGP to prevent calcification of the elastic fibers [8]. However, MGP is synthesized as dp-ucMGP, and needs activation by vitamin K-dependent carboxylation to be able to protect elastic fibers in the extracellular matrix from calcification. These processes could create, or exacerbate, a pre-morbid, vitamin K deficit during severe disease and increased demand of vitamin K. 

Aside from the carboxylation of prothrombotic proteins, vitamin K is also essential for carboxylation of antithrombotic proteins (e.g., Proteins S, Protein C) [12]. The prothrombotic proteins and Protein C are almost exclusively hepatic proteins, whereas approximately 50% of Protein S is extrahepatically synthesized and activated by vitamin K [13], and MGP is primarily synthesized and activated extrahepatically [14]. During a state of vitamin K deficiency, e.g., increased vitamin K use during acute illness, vitamin K is primarily used for carboxylation of the prothrombotic coagulation factors in the liver and, to a lesser extent, for carboxylation of the extrahepatic vitamin K-dependent proteins, including MGP and the antithrombotic Protein S [6]. It has been hypothesized that this could induce a prothrombotic state with increased blood clotting in peripheral tissues, as has been seen in COVID-19 patients [10]. 

We aimed to test the hypotheses that low vitamin K status is a common characteristic of hospitalized patients with COVID-19 compared to population controls and that low vitamin K status predicts mortality in hospitalized COVID-19 patients. 

## 2. Materials and Methods

### 2.1. The Amager Hvidovre Hospital COVID-19 Cohort

Characteristics of the Amager Hvidovre Hospital (Hvidovre) COVID-19 cohort have previously been described [15]. Briefly, this retrospective case series included adults 18 years of age or older with a new-onset pulmonary infiltrate and confirmed SARS-CoV-2 infection who were consecutively admitted between 10 March and 23 April 2020 at a 700-bed university-affiliated hospital in Copenhagen, Denmark. Cases were confirmed through reverse-transcriptase–polymerase-chain-reaction assays performed on an oropharyngeal swab or a lower respiratory tract specimen. Data including patient characteristics, vital parameters and laboratory measurements were transferred from electronic health records. The study was approved by the Danish Patient Safety Authority (record no. 31-1521-309) and the Regional Data Protection Center (record no. P-2020-492). A blood sample was drawn within 4 days from admission. In case more than one sample was drawn from the same patient, the dp-ucMGP measurement in the first sample was used in the statistical analyses. EDTA plasma was separated by centrifugation and stored at minus 80 °C. Measurements of dp-ucMGP in stored plasma samples from the biobank were approved by the Ethical Committee of the Capital Region of Denmark (record no. H-20047597). 

### 2.2. General Population Controls

The population-based Health 2016 [16] study was performed at the Center for Clinical Research and Prevention (CCRP). A total of 4497 randomly selected persons living in 11 municipalities in the Western part of Copenhagen, covering part of the catchment area of Amager Hvidovre Hospital, were invited to a health examination and 1251 (28%) participated. All participants underwent a health examination and completed a questionnaire about lifestyle and health. Measurements of dp-ucMGP were performed in 491 consecutive participants (aged 19–71 years) examined between 30 May 2017 and 21 December 2017 [3]. For the present study population, controls were matched 1:1 to each COVID-19 patient (*n* = 138) by sex and age (closest age) (*n* = 138). The Health 2016 study was approved by the Ethics Committee of the Capital Region of Denmark (record no. H-15017277). 

### 2.3. Measurements of Vitamin K Status in Plasma

In both patients and controls, biochemical determination of vitamin K status was performed by using the IDS-iSYS InaKtif MGP assay (Immunodiagnostic systems, plc, Tyne and Wear, UK) performed at the Department of Clinical Biochemistry, Rigshospitalet, Glostrup, Denmark [3]. The InaKtif MGP assay is an in vitro diagnostic test intended for the quantitative determination of dp-ucMGP in human plasma on the IDS-iSYS Multi-Discipline Automated System. Control specimens had average values of 900 pmol/L, 4100 pmol/L, and 7050 pmol/L with intra-assay CV (coefficient of variation) of 3.9%, 0.9%, and 0.9%, respectively. Inter-assay CVs were 3.2%, 2.8%, and 1.6%, respectively. Since the reportable range is 300–12,000 pmol/L, dp-ucMGP values < 300 pmol/L were fixed as 299 pmol/L. The plasma samples from the Hvidovre COVID-19 cohort were analyzed in October 2020. To examine the stability in the stored samples, we performed repeated measurements of dp-ucMGP in 20 samples from another study after 3 months of storage. The mean at baseline and after 3 months was 392.1 and 396.3 pmol/L, respectively (mean difference 4.2; 95% CI: −14.6; 23.0).

### 2.4. Statistical Analyses

Dp-ucMGP was used as a continuous variable or categorical variable in four categories (25% quartile, 50% quartile, 75% quartile, and above 75% quartile of dp-ucMGP). Dp-ucMGP values were log-transformed (log2) in the analyses and back-transformed when presented. Baseline characteristics were reported as frequencies with percentages, means with SD or medians with interquartile range (IQR). Comparison between COVID-19 patient groups (non-survivor patients vs. survivor patients) was performed using X2 tests, Fisher’s exact test, *t*-test or Mann–Whitney U as appropriate. Kaplan–Meier survival plots were drawn for each of the four dp-ucMGP categories and compared by the log-rank test. Hazard ratios (HRs) with 95% confidence intervals (CIs) were estimated using Cox proportional hazards models with time from blood sampling as the underlying time scale and a doubling of dp-ucMGP as exposure. All Cox proportional hazard models were tested for proportionality using weighted residuals [17]. Patients entered the analysis at the time of blood sampling and were followed until death or 90 days after blood sampling. Thus, the HR estimate reflects the relative risk for death within 90 days per one doubling of dp-ucMGP. The regression survival analyses were further adjusted for sex and age. In all statistical models, users of vitamin K antagonists (shown in Table 1) were excluded. Age, BMI, and eGFR were included as continuous variables. Estimated Glomerular Filtration Rate (eGFR) was calculated as described by Selistre et al. [18]. Imputation of values of BMI and eGFR, when missing, was performed using multiple imputation with 15 iterations. Predictors for the multiple imputation were the following variables: age, sex, diabetes, hypertension, CVD, and asthma/chronic obstructive pulmonary disease. A *p*-value < 0.05 was considered significant. Data analysis was performed using R software version 4.0.2 (R Foundation for Statistical Computing, Vienna, Austria).

## 3. Results

**Figure 1:**
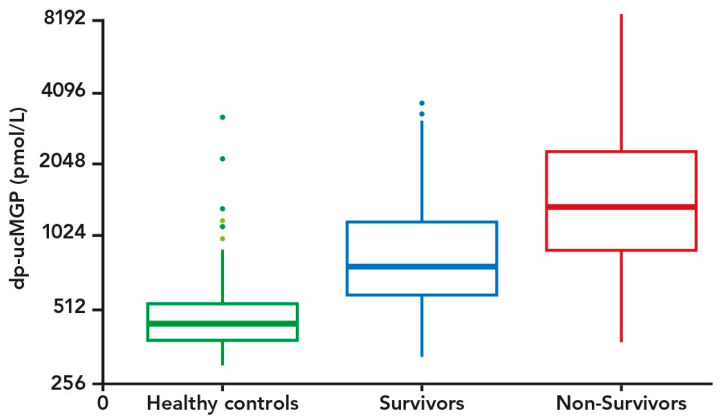
Boxplot of dp-ucMGP levels (pmol/L) among controls (*n* = 138) and COVID-19 patients (Survivors (*n* = 43) and Non-Survivors (*n* = 95)). The *y*-axis is on an exponential scale.

Dp-ucMGP was measured in a total of 138 COVID-19 patients. Thirty-six and 43 died within 30 and 90 days from inclusion, respectively. Table 1 shows characteristics of the COVID-19 patient cohort stratified by 90-day survival status. Four patients and two controls were recorded as users of vitamin K antagonists, and they were all excluded from subsequent analyses. Among the patients, both 90-day mortality and 30-day mortality (data not shown) were significantly associated with high age, hypertension, cardiovascular disease (CVD), and increased levels of dp-ucMGP. Furthermore, levels of dp-ucMGP were higher among COVID-19 patients compared to the population controls (Table 1). Only three patients were recorded to have developed venous thromboembolic complications during hospitalization. A boxplot of levels of dp-ucMGP in survivor and non-survivor patients and controls is shown in Figure 1. The Cox regression analysis showed that the mortality risk was significantly higher in patients with increased levels of dp-ucMGP (unadjusted HR per doubling of dp-ucMGP was 1.95, 95% CI: 1.41; 2.69). The HR estimate attenuated after adjusting for sex and age (sex and age adjusted HR per doubling of dp-ucMGP was 1.49, 95% CI: 1.03; 2.24). The HR attenuated and became statistically insignificant after additional adjustment for CVD, diabetes, and BMI (sex, age, CVD, diabetes, and BMI adjusted HR per doubling of dp-ucMGP was 1.29, 95% CI: 0.87; 1.92). After additional adjustment for eGFR, the HR attenuated slightly more (sex, age, CVD, diabetes, BMI, and eGFR adjusted HR per doubling of dp-ucMGP was 1.22, 95% CI: 0.82; 1.80).

When ending follow-up at 30 days from blood drawing, the mortality risk was 1.97 (unadjusted HR, 95% CI: 1.41; 2.77) and 1.53 (sex and age adjusted HR, 95% CI: 1.04; 2.24). The HR attenuated and became statistically insignificant after additional adjustment for CVD, diabetes, and BMI (sex, age, CVD, diabetes, and BMI adjusted HR per doubling of dp-ucMGP was 1.21, 95% CI: 0.80; 1.84). After additional adjustment for eGFR, the HR attenuated slightly more (sex, age, CVD, diabetes, BMI, and eGFR adjusted HR per doubling of dp-ucMGP was 1.16, 95% CI: 0.77; 1.75).

**Figure 2:**
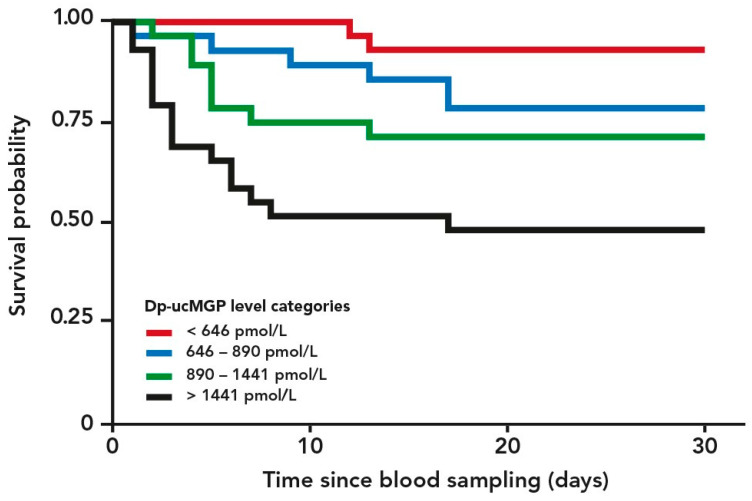
Kaplan–Meier plot of 90-day cumulated risk of dying versus time from blood drawing stratified by levels of dephosphorylated uncarboxylated Matrix Gla Protein (dp-ucMGP) categories (quartiles of dp-ucMGP). High levels of dp-ucMGP reflect low vitamin K status. *p*-value for log-rank test for comparison of the groups was <0.0001.

A Kaplan–Meier plot of cumulated risk of dying versus time from blood drawing stratified by dp-ucMGP categories (lowest quartile, two middle quartiles, and highest quartile) is shown in Figure 2. The log-rank test for comparison of the four dp-ucMGP categories (quartiles) was highly statistically significant (*p* < 0.0001). 

**Figure 3:**
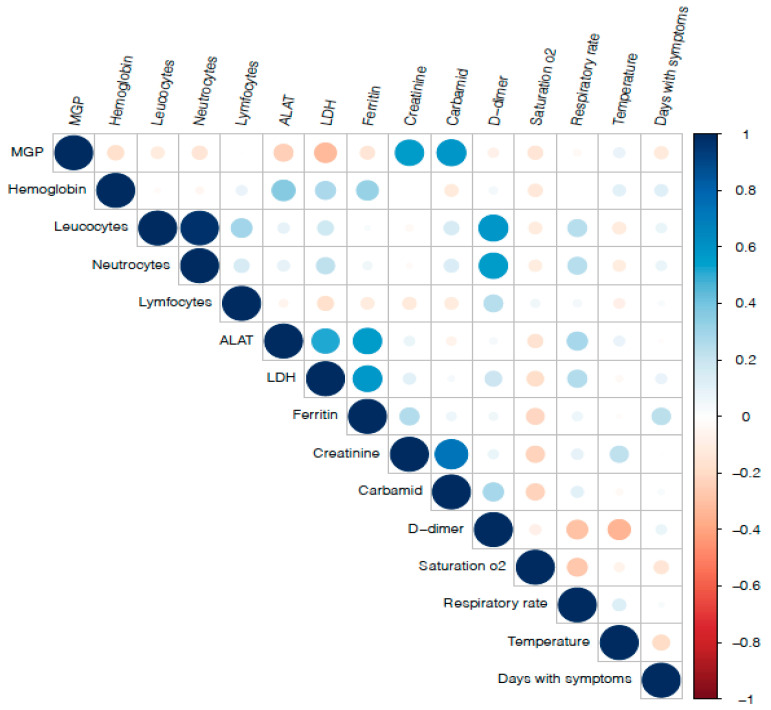
A correlation matrix of measured blood biomarkers and clinical parameters in hospitalized COVID-19 patients. Blue and red colored circles represent positive and negative correlations, respectively. The size of circles reflects the strength of the correlations.

A correlation matrix of a selection of measured blood biomarkers and clinical parameters is presented in Figure 3. Dp-ucMGP was positively associated with plasma creatinine (0.56, *p* < 0.0001) and urea (0.58, *p* < 0.0001) and inversely with alanine aminotransferase (−0.25, *p* = 0.01) and lactate dehydrogenase (−0.33, *p* = 0.002). All other correlations were statistically insignificant.

## 4. Discussion

Our results showed that vitamin K status is lower in hospitalized patients with COVID-19 compared to population controls and that low vitamin K status predicts higher mortality among patients with COVID-19. These findings suggest that vitamin K could play a role in the disease mechanisms in COVID-19. 

SARS-CoV-2 infection may also lead to hypercoagulability and thrombosis [19,20] in some patients undergoing high-dose anticoagulative treatment [21]. The pattern of thrombotic events appears to be different compared with severe pneumonia caused by influenza [22]. It may appear somewhat surprising that COVID-19 patients, who seem prone to thromboembolism, have lower vitamin K status, since lowering vitamin K function pharmaceutically by vitamin K antagonists is commonly used as an antithrombotic treatment and for the prevention of thromboembolic events in high-risk patients. A possible explanation for this apparent paradox may be that during a state of severe vitamin K deficiency, the intrahepatic vitamin K-dependent carboxylation (activation) of prothrombotic proteins is prioritized, or preserved, at the expense of peripheral activation of vitamin K-dependent proteins such as the antithrombotic Protein S and the calcification inhibitory MGP. This is supported by the earlier finding of a preserved intrahepatic prothrombotic effect, as reflected by normal levels of PIVKAII, in COVID-19 patients in spite of significantly increased levels of dp-ucMGP [6]. In addition, the decreased activation of the calcification inhibitory MGP may increase calcification and subsequent degradation of elastic fibers in the extracellular matrix of lung tissue, leading to more severe lung damage in COVID-19 patients. 

As countries worldwide are experiencing a second or even third wave of the COVID-19 pandemic, there is an urgent need for measures to improve the outcome and long-term consequences of COVID-19. Supplementation with vitamin K represents an inexpensive and simple-to-use add-on to other treatments, and it would therefore be interesting to explore whether vitamin K supplementation in addition to other treatment can improve the outcome of COVID-19. It is of potential interest that obesity is a predictor of poor outcome of SARS-CoV-2 infection [23]. This could be in line with our recent report that obesity was strongly associated with higher levels of dp-ucMGP (indicating low vitamin K status), providing a possible explanation for the link between obesity and COVID-19 [3]. 

There are several limitations in the present study. Firstly, the observational study design does not allow us to draw definite conclusions regarding causality. Randomized trials are needed to document the potential beneficial effects of vitamin K supplementation on the course of COVID-19 disease. Second, high levels of dp-ucMGP have been associated with other conditions, e.g., cardiovascular disease and diabetes, which are also associated with poor outcomes of COVID-19, and could act as confounders. It is noteworthy that low kidney function (eGFR) was associated with low vitamin K status (high dp-ucMGP) in our patient population and adjustment for renal function did cause attenuation of the association between vitamin K status and mortality. This may suggest that impaired kidney function is involved in the disease mechanisms linking vitamin K to poor outcome of COVID-19. Alternatively, the low vitamin K status and COVID-19 association could be confounded, and fully explained, by impaired kidney function. The size of the present study did not allow us to perform statistical models including all variables of potential interest. The causal relation between vitamin K status and COVID-19, and the causal pathway, should be explored further in other studies of sufficient size to allow for full adjustment for confounders and explore causal pathways. Third, we did not have data on vitamin D status such as blood levels of 25-OH vitamin D. This is a limitation because it has been hypothesized that vitamin D and K could interact in COVID-19 disease [10]. Fourth, we do not have measurements of total MGP, and dp-ucMGP levels are therefore not corrected for total MGP. It is a limitation that we only have measured one biomarker of vitamin K status. It is of potential interest that increased levels of another vitamin K-dependent protein and biomarker of vitamin K status, plasma growth arrest-specific factor 6 (GAS6), have been found to be associated with disease severity and mortality in hospitalized COVID-19 patients [24].

Fifth, assessment of lung damage such as CT-scans would have added valuable information on the tissue-specific effects of vitamin K deficiency during COVID-19. Finally, a long-term follow-up of persistent symptoms among survivors of COVID-19 would have been of great interest to investigate, since any measure that prevents short-term outcomes may also have an influence on long-term outcomes such as persistence of symptoms. 

In conclusion, in the present study, we confirmed that vitamin K status is markedly lower in hospitalized COVID-19 patients compared to population controls and that low vitamin K status was associated with mortality in patients with COVID-19 in age- and sex-adjusted analyses. However, the association between vitamin K status and mortality attenuated and became statistically insignificant after adjustment for co-morbidities, suggesting that co-morbidities could be part of the causal pathway or confounders of the association of vitamin K status with mortality. Whether vitamin K supplementation in COVID-19 patients can change the course of disease and prevent death or long-term consequences of COVID-19 remains to be tested in randomized clinical trials.

## Figures and Tables

**Table 1:** Characteristics of the COVID-19 patient cohort survivors and non-survivors, and general population controls.

		COVID-19 Non-survivor Patients (*n* = 43)	COVID-19 Survivor Patients (*n* = 95)	*p*-Value #	COVID-19 Patients Total (*n* = 138)	Population Controls (*n* = 138)	*p*-Value *
Age	Mean (SD)	76.6 (11.5)	64.7 (15.8)	<0.0001	68.4 (15.6)	62.5 (10.6)	0.0002
Sex	Male	19 (44.2)	40 (42.1)		59 (42.8)	59 (42.8)	1
	Female	24 (55.8)	55 (57.9)	0.9656	79 (57.2)	79 (57.2)	
BMI	mean (SD)	29.2 (6.5)	28.8 (6.8)	0.7244	28.9 (6.6)	26.5 (5.3)	0.0016
Obesity	BMI ≥ 30	15 (41.7)	31 (37.8)		46 (39.0)	23 (16.7)	0.0001
	BMI < 30	21 (58.3)	51 (62.2)	0.8484	72 (61.0)	115 (83.3)	
Hypertension	No	15 (34.9)	57 (60.0)		72 (52.2)	45 (32.6)	0.0015
	Yes	28 (65.1)	38 (40.0)	0.0107	66 (47.8)	93 (67.4)	
Diabetes	No	28 (65.1)	68 (71.6)		96 (69.6)	129 (93.5)	<0.0001
	Yes	15 (34.9)	27 (28.4)	0.5724	42 (30.4)	9 (6.5)	
CVD	No	13 (30.2)	48 (50.5)		61 (44.2)	106 (90.6)	<0.0001
	Yes	30 (69.8)	47 (49.5)	0.0415	77 (55.8)	11 (9.4)	
Asthma/COPD	No	32 (74.4)	78 (82.1)		110 (79.7)	99(85.3)	0.3141
	Yes	11 (25.6)	17 (17.9)	0.4171	28 (20.3)	17(14.7)	
Vitamin K antagonist user	No Yes	41 (95.3) 2 (4.7)	93 (97.9) 2 (2.1)	0.7811	134 (97.1) 4 (2.9)	136 (98.6) 2 (1.4)	0.6798
eGFR (mL/min/1.73 m^2^)	Mean (95% CI)	47.2 (41.6; 52.8)	65.6 (61.1; 70.1)	<0.0001	60.7 (56.7; 64.7)	81.1 (78.6; 83.6)	<0.0001
Dp-ucMGP (pmol/L)	Median (IQR)	1368 (889; 2340)	771 (590; 1174)	0.0002	889 (646; 1441)	506.5 (417.0; 617.2)	<0.0001
Dp-ucMGP (pmol/L)	Mean (95% CI)	1445 (1148; 1820)	877 (778; 995)	<0.0001	1022 (912; 1151)	533 (498; 571)	<0.0001

BMI: body mass index; CVD: cardiovascular disease; COPD: chronic obstructive pulmonary disease; IQR: interquartile range; SD: standard deviation; Dp-ucMGP: Dephosphorylated and uncarboxylated Matrix Gla Protein; eGFR: estimated Glomerular Filtration Rate. # *p*-value for comparison of non-survivor and survivor patients using X2 tests, Fisher’s exact test, *t*-test, or Mann–Whitney U test as appropriate. * *p*-value for comparison of patients (*n* = 138) and sex- and age-matched controls (138) using X2 tests, Fisher’s exact test, *t*-test, or Mann–Whitney U test as appropriate.

## Data Availability

The data presented in this study are available on request from the corresponding author. The data are not publicly available due to Danish regulations on personal data protection.
